# A retrospective study on the impact of different antibiotic regimens in non-surgical periodontal therapy on microbial loads and therapy outcomes

**DOI:** 10.3389/froh.2025.1578484

**Published:** 2025-05-14

**Authors:** Nils Werner, Vinay Pitchika, Katrin Heck, Christina Ern, Richard Heym, Ali Al-Ahmad, Fabian Cieplik, Falk Schwendicke, Caspar Victor Bumm, Matthias Folwaczny

**Affiliations:** ^1^ Department of Conservative Dentistry and Periodontology, LMU University Hospital, LMU Munich, Munich, Germany; ^2^ Private Practice, Munich, Germany; ^3^ Department of Operative Dentistry and Periodontology, Center for Dental Medicine, Medical Center & Faculty of Medicine, University of Freiburg, Freiburg, Germany

**Keywords:** periodontal therapy, periodontitis, antibiotics, antibiotic resistance, periodontal bacteria

## Abstract

**Introduction:**

This study aimed to analyse the impact of different antibiotic regimens during non-surgical periodontal therapy on the microbial load of selected periodontitis-associated bacteria (PAB) and the primary therapy outcomes.

**Methods:**

For this aim, 259 patients received steps I and II of periodontal therapy and were included in this clinical trial. 202 patients were treated without the adjunctive use of systemic antibiotics, 18 received amoxicillin (AMOX) as well as metronidazole (MET) and 39 only MET. Subgingival biofilm samples were quantitatively analysed for selected PAB using DNA-DNA-hybridisation-based detection assays for microbial loads of PAB before and 6 months after treatment. Changes in the microbial load of PAB and achievement of a “treat-to-target” endpoint (T2T) (≤4 sites with probing depth ≥5 mm) were analysed. Patients' subgingival microbial load was significantly reduced following therapy.

**Results:**

38.2% of the patients achieved T2T. Binary logistic regression adjusted for confounders indicated a relationship between residual PAB levels and not achieving T2T. In patients not receiving systemic antibiotics a 2.4-fold increased risk of not reaching T2T after steps I and II therapy was observed (none vs. MET aOR = 2.38 *p* = 0.44). Linear regression analysis adjusted for T0 PAB concentration and confounders revealed an increased reduction of PAB levels in patients with systemic antibiotics. No difference in PAB reduction or chance of achieving T2T was observed between MET and MET + AMOX.

**Discussion:**

Microbial loads of PAB were found directly associated with periodontal status. As antibiotic treatment with both MET and MET + AMOX similarly reduced microbial loads of PAB, treatment with MET alone may be sufficiently effective as adjunctive to non-surgical periodontal treatment. To confirm this, further prospective studies with bigger sample size are needed.

## Introduction

The onset of periodontitis involves a dysbiotic shift in the subgingival microbiota, along with an exaggerated immune-inflammatory infiltration within the periodontium that is largely host-specific (1, 2). The role of subgingival microbiota in periodontitis is multifaceted; however, alterations in the composition of the subgingival biofilm, as well as an increase in microbial load, are associated with periodontitis (3, 4).

Steps I and II of periodontal therapy include a pre-procedure consultation, oral hygiene instruction, professional mechanical plaque reduction (PMPR) and subgingival instrumentation of periodontally compromised teeth (5). Accordingly, steps I and II therapy are directed towards the elimination of both inflammation and resolution of dysbiosis in the subgingival microbiota. Together both steps are recommended as a first-line treatment (6–10).

Generally, the success of individual therapies is difficult to predict and varies greatly from patient to patient (11–13). Particularly for the treatment of young patients with rapidly progressing disease, prescriptions of various systemic antibiotics are suggested as adjunctive to primary therapy. Especially, the combination of amoxicillin and metronidazole (AMOX + MET) has been suggested by and investigated in numerous studies indicating additional pocket reduction (14–17). In addition, early studies by Loesche et al. showed that metronidazole alone had a positive effect on the outcome of periodontal therapy. The results highlighted a significant reduction in the need for periodontal surgery and a significantly better reduction in anaerobic species than with a placebo adjuvant in the treatment of stages I and II (18–20).

Antimicrobial resistance (AMR) presents a major concern for global health care, with an estimated number of deaths due to AMR expected to rise to 10 million per year by 2050 (21). In this context, the oral cavity may act as a significant reservoir for AMR (22–25), which is also reflected by several policy statements on AMR in 2024 and 2025 by relevant stake holders such as WHO, FDI and IADR (26). Concerning periodontitis-associated bacteria (PAB), resistance to AMOX and clindamycin has increased in recent years (27). For this reason, guidelines for the treatment of stage I–III periodontitis handle the use of antibiotics restrictively (5). Interestingly, Janina Lewis's lab has shown promising results with amixicile, a newer antibiotic with a similar spectrum of activity to metronidazole, with fewer systemic side effects and lower risk of resistance (28, 29).

In the past, various strategies for prescribing antibiotics in periodontal therapy have been discussed. The most commonly accepted approaches recommend adjunctive antibiotics according to the age of the patient, the severity of the disease and the detection of specific PAB (30, 31). Yet, due to the high complexity of the subgingival microbiome, the presence of PAB as rationale for the use of adjunctive antibiotics might be too simplified (3, 32, 33). Additionally, these tests showed insufficient inter- and intra-test reproducibility (34).

The present study aimed to retrospectively analyse the impact of different antibiotic regimens during steps I and II therapy on the microbial load of selected PAB collected from subgingival biofilm on the primary therapy outcome.

## Materials and methods

### Study design and source of data

This retrospective study was approved by the Ethics Committee of the Medical Faculty of the Ludwig-Maximilians-University (LMU), Munich, Germany (No. 022-0669) and complies with the ethical principles proposed by the Declaration of Helsinki. The study is registered at the German Clinical Trials Register (DRKS00028923). The study description follows the guidelines for Strengthening and Reporting in Observational Studies (STROBE) (35).

### Study population

This study observed 259 patients, who were enrolled into steps I and II therapy for the treatment of periodontitis in the undergraduate course at the Department of Conservative Dentistry and Periodontology, University Hospital, LMU Munich between February 2011 and March 2016 (10). All subjects enrolled in the study received the first two steps of therapy upon initial diagnosis of periodontitis or diagnosis of recurrent periodontal disease. To be eligible for inclusion in the study, patients had to fulfill the following criteria: (1) Age ≥18 and ≤80 years, (2) Diagnosis of stage III or IV periodontitis according to the current classification (36), (3) Periodontal charting before (T0) and after (T1) the first two steps of therapy with documentation of probing pocket depth (PPD) and bleeding on probing (BOP) at six sites per tooth (4), Laboratory analysis of subgingival biofilm at T0 and T1, assessing six PAB: *Porphyromonas gingivalis, Aggregatibacter actinomycetemcomitans, Prevotella intermedia, Fusobacterium nucleatum, Treponema denticola*, and *Tannerella forsythia.* Non-inclusion criteria were: (1) Pregnancy, (2) Previous periodontal treatment within 2 years prior to study enrolment, (3) Current participation in supportive periodontal care (SPC), (4) Conditions associated with a temporary or permanent impairment of immune function.

### Periodontal treatment

Periodontal treatment was described earlier in detail (10). In brief, all patients received thorough education on the causes, development, risk factors, and treatment options for periodontitis. Step I included personalized oral hygiene instruction and PMPR. Teeth with PPD of more than 3 mm were treated with subgingival instrumentation under local anesthesia using the SonicFLEX device (KaVo Dental, Biberach, Germany) and Gracey curettes (Hu-Friedy, Chicago, USA) without any time limitation (10, 11). Adjunctive antibiotics were prescribed in cases with advanced periodontitis or the detection by a commercial microbial test of specific PABs, in particular *A. actinomycetemcomitans*. All patients adhering to the indication for adjunctive antimicrobial therapy received 400 mg MET three times a day, while patients who were classified as *A. actinomycetemcomitans* positive received an additional 500 mg AMOX three times a day, all for 7 days (17).

### Sample collection and analysis

The procedure for subgingival biofilm collection was performed by CE and RH. PAB analysis has been described in detail previously (37). In brief, samples were collected from the deepest pockets in each quadrant using sterile paper points and pooled for analysis. To monitor specific changes in microbial load, the same sites were sampled at both T0 and T1. Bacterial DNA was extracted using the MagNA Pure DNA Isolation Kit III (Roche Diagnostics, Mannheim, Germany) according to the manufacturer's protocol. DNA amplification was performed using the Parident-kit (AMPLEX Diagnostics, Gars am Inn, Germany), in which 5 µl of each DNA sample was combined with 45 µl of the bacteria-specific master mix. Hybridisation-based detection assay was then performed, with each sample transferred to colour-coded wells for six target bacteria. After incubation with hybridisation buffer, peroxidase conjugate was added, followed by the chromogenic substrate 3, 3′, 5, 5′-tetramethylbenzidine for detection. Changes in optical density (OD) were measured at 450 nm and 620 nm using a Varioskan spectrophotometer (Thermo Fisher Scientific, Waltham, USA).

### Clinical parameters

Periodontal examination was conducted prior to steps I and II therapy (T0) and after 6 months (T1) (10). PPD was measured to the nearest millimetre using a PCP-12 periodontal probe with a trained probing force of 0.2–0.3 N (38). All measurements were reassessed by RH and CE. Afterwards the findings were compared with the students' and all probing depths that differed by at least 2 mm were remeasured and consensus was found. BOP was determined approximately 30 s after probing (39). Mobility was assessed according to Miller (40). Furcation involvement (FI) was measured with a 2N-Nabers probe and graded as described by Hamp et al. (41). Individual periodontal diagnosis was based on the 2018 classification (36). At the site level proportions of periodontal pockets (PPD%) were calculated at T0 and T1 using the parameter pocket closure defined per site, as a PPD of 4 mm in the absence of BOP or ≤3 mm, as stated by the latest classification (42). The proportion of sites with deep periodontal pockets was calculated based on the threshold PPD >5 mm. Smoking status is defined as current smoking or non-smoking. As the primary outcome, the treat-to-target (T2 T) endpoint suggested by Feres et al. as ≤4 sites with PPD ≥5 mm was used (43).

### Power analysis

A formal *a priori* sample size calculation was not feasible given the retrospective nature of the study. For this reason, *post hoc* power was calculated using G*Power (version 3.1), based on the R Square values obtained from the linear regression model of difference in *P. gingivalis* levels (0.363). This analysis evaluates the overall effect of antibiotic regimens on microbial load. Using standard practice for multiple regression models, additional parameters were the number of predictors, a significance level of 0.05, and the total sample size of the study, not taking into account differences in group sizes. The calculated power for the study was 1.00, which is well above the commonly accepted threshold of 0.80.

### Statistical analysis

Numerical data are expressed as mean (±SD), categorical variables as absolute and relative frequencies (percentages). Non-normally distributed variables are presented as median and interquartile range [q1; q3]. Normality of data was determined using the Kolmogorov–Smirnov test. For descriptive analysis, differences were compared using Student's *t*-test or analysis of variance for continuous variables, Mann–Whitney *U* test or Kruskal–Wallis test for ordinal and skewed variables, and Chi-squared test for categorical variables. Linear regression was used to model PAB changes over therapy, adjusting for confounding variables T0 PAB OD, sex, diabetes and smoking status. Adjusted beta coefficients and 95% confidence intervals (95% CI) are reported as effect estimates for changes in PAB concentration. Logistic regression models were used to identify potential confounders of T2T. For the association between T1 PAB level and T2T, a binary logistic regression model was used including each PAB and potential confounders (sex, diabetes and smoking). For the association between T2T and antibiotic use, a binary logistic regression model was calculated including antibiotic medication and possible confounders (T0 PPD%, sex, diabetes and smoking status). Results are presented as adjusted odds ratios (aOR) per 1 unit change in PC with corresponding 95% (CIs). The significance level of *α* = 0.05 was applied for all tests. Model quality was described using Nagelkerke *R*^2^ for binary and *R* Square values for linear models. All analyses were performed with SPSS (version 29.0, IBM, Armonk, USA).

### Source of bias

Periodontal diagnosis and treatment were conducted as part of the undergraduate program at the Department of Conservative Dentistry and Periodontology, University Hospital of LMU Munich. All steps of therapy and diagnosis were supervised and controlled by CE and RH calibrated for periodontal probing in advance (inter-rater reliability of periodontal probing, kappa = 0.82) (44, 45).

## Results

### Patient and periodontal characteristics

**Figure 1:**
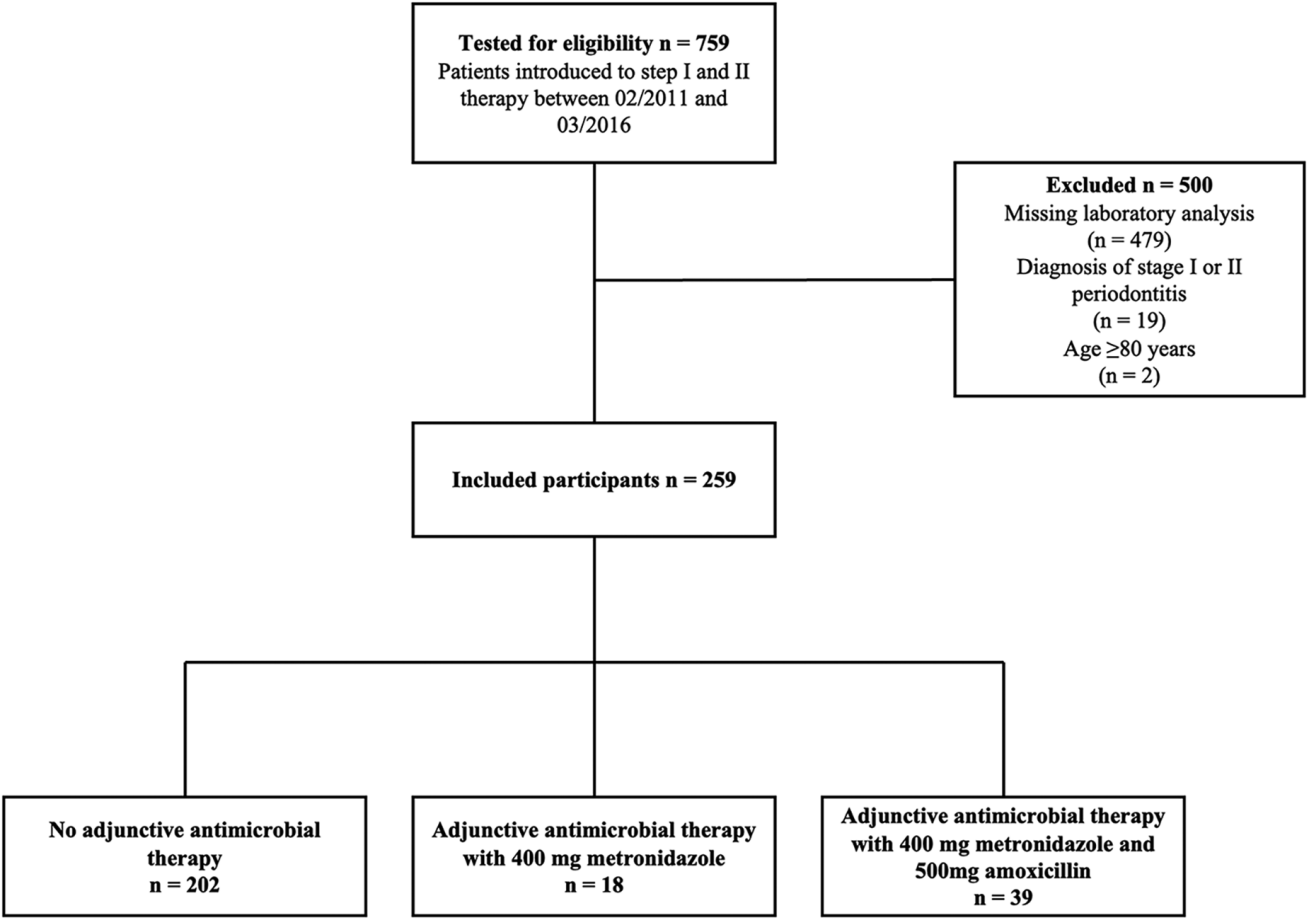
Flow diagram of the process of trial inclusion/exclusion.

Seven hundred fifty-nine patients received steps I and II therapy between February 2011 and March 2016. The final analysis included 259 patients showing a median age of 60 years (Figure 1). The male-to-female ratio was 55.6/44.4%, 27.8% of study subjects were current smokers, and 8.9% had been diagnosed with diabetes mellitus (Table 1). Patients presented with periodontal pockets at 21% [12%; 34%] of all sites and with deep periodontal pockets at 4% [1%; 9%] of all sites at T0. When classifying patients retrospectively 206 (79.5%) were assigned to stage III and 53 (20.5%) to stage IV. Among the 230 patients eligible for grading 115 (52.3%) were classified as grade B and 105 (47.7%) as grade C (Table 1).

**Table 1:** Baseline characteristics.

Variable	Total (*n* = 259)	No systemic antibiotics (*n* = 202)	AMOX + MET (*n* = 18)	MET (*n* = 39)	*p*-value
Age, y	60 [51; 69]	62 [52; 69]	60 [51; 70]	57 [46; 67]	0.109
Female, *n* (%)	115 (44.4)	84 (41.6)	8 (44.4)	23 (59.0)	0.135
Male, *n* (%)	144 (55.6)	118 (58.4)	10 (55.6)	16 (41.0)	
Non-diabetic, *n* (%)	254 (90.7)	201 (90.5)	16 (88.9)	37 (92.5)	0.914
Diabetic, *n* (%)	23 (8.9)	18 (8.9)	2 (11.1)	3 (7.7)	
Non-smokers, *n* (%)	187 (72.2)	146 (72.3)	14 (77.8)	27 (69.2)	0.798
Smokers, *n* (%)	72 (27.8)	56 (27.7)	4 (22.2)	12 (30.8)	
<10 cigarettes per day	19 (26.4)	14 (25.0)	2 (50.0)	3 (25.0)	0.388
≥10 cigarettes per day	53 (73.6)	42 (75.0)	2 (50.0)	9 (75.0)	
Number of teeth per patient, *n*	24 [19; 27]	24 [19; 27]	23 [15; 27]	24 [18; 25]	0.744
Stage					
III, *n* (%)	206 (79.5)	163 (72.2)	13 (72.2)	30 (79.5)	0.631
IV, *n* (%)	53 (20.5)	39 (27.8)	5 (27.8)	9 (23.1)	
Grade					
B, *n* (%)	115 (52.3)	99 (58.6)	8 (53.3)	8 (22.2)	**<0**.**001**
C, *n* (%)	105 (47.7)	70 (41.4)	7 (46.7)	28 (77.8)	

AMOX, amoxicillin; MET, metronidazole.

Continuous data are presented as median [q1; q3]; nominal data are presented as *n* (%).

Comparisons are performed using Kruskal–Wallis test or Chi-Square test.

Bold indicates statistically significant values (*P* < 0.05).

Among the 259 patients, 57 received systemic antibiotics during step II therapy (*n* = 39 MET *n* = 18 AMOX + MET). These patients were retrospectively classified with a higher grade of disease progression (*p* < 0.001) (Table 1).

When looking at the periodontal status at time T0, it is evident that patients who received an antibiotic as adjunctive for steps I and II therapy had significantly more severe periodontal disease than patients who did not. In particular, there was a significantly higher proportion and number of all and deep periodontal pockets. However, the antibiotic groups did not differ in this comparison (Table 2).

**Table 2:** Periodontal characteristics.

Variable	Total (*n* = 259)	No systemic antibiotics (*n* = 202)	AMOX + MET (*n* = 18)	MET (*n* = 39)	*p*-value
Full mouth bleeding score at T0, %	31 [21; 50]	31 [21; 50]	43 [30; 63]	31 [17; 51]	0.060
Full mouth bleeding score at T1, %	23 [13; 38]	25 [14; 39]	23 [15; 46]	17 [8; 32]	**0** **.** **047**
Full mouth plaque score at T0, %	31 [18; 44]	32 [19; 44]	32 [15; 47]	25 [9; 39]	0.148
Full mouth plaque score at T1, %	46 [29; 64]	46 [29; 63]	50 [25; 72]	44 [27; 68]	0.851
Proportion of sides with periodontal pockets at T0, %	21 [12; 34]	17 [10; 28]	38 [27; 53]	31 [24; 41]	**<0.001** ^a^ ^,^ ^b^
Proportion of sides with deep pockets at T0, %	4 [1; 9]	3 [0; 6]	12 [6; 21]	12 [7; 21]	**<0.001** ^a^ ^,^ ^b^
Proportion of sides with periodontal pockets at T1, %	9 [5; 18]	9 [5; 18]	10 [3; 27]	8 [5; 27]	0.977
Proportion of sides with deep pockets at T1, %	1 [0; 3]	1 [0; 3]	3 [0; 5]	1 [0; 5]	0.878
Proportional reduction of sites with periodontal pockets, %	53 [30;75]	48 [24; 69]	78 [54; 90]	75 [44; 90]	**<0.001** ^a^ ^,^ ^b^
Proportional reduction of sites with deep pockets, %	67 [36; 96]	59 [28; 88]	89 [59; 100]	87 [68;100]	**<0.001** ^a^ ^,^ ^b^
Endpoint reached, *n* (%)	99 (38.2)	75 (37.1)	9 (50.0)	15 (38.5)	0.560

AMOX, amoxicillin; MET, metronidazole, T0, prior to steps I and II therapy; T1, after steps I and II therapy.

Continuous data are presented as median [q1; q3]; nominal data are presented as *n* (%).

Comparisons are performed using Kruskal–Wallis test or Chi-Square test.

Bold indicates statistically significant values (*P* < 0.05).

^a^ None vs. AMOX + MET < 0.05.

^b^ None vs. MET < 0.05.

### Periodontal infection and therapy outcome

All patients showed an improved periodontal status at the time of T1. This was demonstrated by decreasing probing depths and a proportional reduction in both deep and overall periodontal pockets. A comparison of the groups reveals comparable outcomes following therapy, despite the presence of more severe periodontitis at T0 in the antibiotic patients. This is further supported by the significantly greater relative pocket reduction observed in patients receiving systemic antibiotics [None: 48% [24%; 69%]; AMOX + MET: 78% [54%; 90%]; MET: 75% [44%; 90%], *p* < 0.001] (Table 2).

Patients within all treatment groups presented with almost similar T0 PAB levels. Six months after therapy, lower *A. actinomycetemcomitans* and *P. gingivalis* levels were observed in patients who received systemic antibiotics (Table 3). Furthermore, a higher reduction of *P. gingivalis, F. nucleatum, T. forsythia* and *T. denticola* between both appointments could be measured in patients receiving systemic antibiotics (Table 3). At the same time, no differences between MET alone and AMOX + MET were observed.

**Table 3:** Optical density of different pathogens at T0 and T1.

Variable	Total (*n* = 259)	No systemic antibiotics (*n* = 202)	AMOX + MET (*n* = 18)	MET (*n* = 39)	*p*-value
T0					
*Aa*	0.05 [0.02; 0.11]	0.05 [0.03; 0.11]	0.03 [0.02; 0.45]	0.05 [0.02; 0.13]	0.911
*Pg*	2.87 [0.34; 4.02]	2.78 [0.19; 4.02]	1.87 [0.47; 3.58]	3.76 [1.11; 4.30]	0.152
*Fn*	1.10 [0.37; 2.08]	1.03 [0.36; 1.95]	1.25 [0.47; 1.92]	1.28 [0.37; 2.55]	0.756
*Pi*	0.17 [0.07; 0.53]	0.20 [0.08; 0.63]	0.11 [0.06; 0.40]	0.10 [0.02; 0.37]	0.100
*Tf*	0.21 [0.08; 0.53]	0.23 [0.08; 0.55]	0.20 [0.09; 0.56]	0.20 [0.10; 0.55]	0.942
*Td*	0.61 [0.08; 1.43]	0.59 [0.01; 1.43]	0.62 [0.25; 1.59]	0.64 [0.06; 1.28]	0.807
T1					
*Aa*	0.05 [0.03; 0.12]	0.06 [0.03; 0.14]	0.03 [0.03; 0.07]	0.04 [0.03; 0.07]	**0** **.** **026**
*Pg*	3.00 [0.32; 4.07]	3.32 [0.53; 4.14]	0.04 [0.02; 3.03]	2.12 [0.51; 3.68]	**0**.**008**^a^
*Fn*	1.49 [0.47; 2.69]	1.60 [0.51; 2.81]	1.36 [0.59; 2.17]	0.83 [0.14; 2.47]	0.100
*Pi*	0.24 [0.10; 0.79]	0.25 [0.10; 0.82]	0.24 [0.10; 0.96]	0.20 [0.11; 0.49]	0.647
*Tf*	0.25 [0.07; 0.85]	0.33 [0.09; 0.99]	0.26 [0.05; 0.51]	0.15 [0.04; 0.55]	0.073
*Td*	0.40 [0.07; 1.52]	0.49 [0.07; 1.67]	0.12 [0.04; 0.82]	0.26 [0.07; 1.10]	0.073
Difference					
*ΔAa*	0.00 [−0.05; 0.03]	−0.01 [−0.06; 0.03]	0.00 [−0.04; 0.42]	0.01 [−0.02; 0.05]	0.193
*ΔPg*	−0.01 [−1.40; 1.13]	−0.10 [−1.43; 0.78]	0.31 [−0.04; 2.38]	0.44 [−1.31; 3.01]	**0**.**011**^b^
*ΔFn*	−0.22 [−1.43; 0.60]	−0.35 [−1.54; 0.50]	−0.43 [−1.34; 0.77]	0.13 [−0.84; 1.31]	0.100
*ΔPi*	−0.03 [−0.29; 0.13]	−0.02 [−0.28; 0.14]	−0.13 [−0.79; 0.01]	−0.05 [−0.17; 0.19]	0.201
*ΔTf*	−0.02 [−0.38; 0.15]	−0.05 [−0.49; 0.13]	−0.05 [−0.13; 0.24]	0.02 [−0.14; 0.24]	0.071
*ΔTd*	0.00 [−0.49; 0.58]	−0.00 [−0.61; 0.40]	−0.21 [−0.04; 0.89]	0.14 [−0.34; 1.00]	**0**.**046**

AMOX, amoxicillin*; Aa*, *Aggregatibacter actinomycetemcomitans*; Δ*,* difference between T0 and T1; *Pg*, *Porphyromonas gingivalis*; *Pi*, *Prevotella intermedia*; *Fn*, *Fusobacterium nucleatum*; MET, metronidazole; T0, prior to steps I and II therapy; T1, after steps I and II therapy; *Td*, *Treponema denticola*; *Tf*, *Tannerella forsythia.*

Data are present as median [q1; q3].

Comparisons are performed using Kruskal–Wallis test and for *post-hoc* Dunn–Bonferroni-Test.

Bold indicates statistically significant values (*P* < 0.05).

^a^ None vs. AMOX + MET < 0.05.

^b^ None vs. MET < 0.05.

Linear regression analysis corroborated these findings concerning a higher reduction of the microbial load in patients with systemic antibiotics. Using a linear regression model for each Δbacteria OD adjusted for T0 PAB level, sex, smoking and diabetes, a potential association between a higher PAB reduction and the use of systemic antibiotics was found [Δ*T. denticola*: β: −0.25(−0.47; 0.003), *p* = 0.034 and Δ*T. forsythia*: β: −0.42 (−0.82; −0.03), *p* = 0.026]. However, no differences between MET and AMOX + MET were found (Table 4). Furthermore, it was observed that patients who reached T2 T had a lower microbial load of PAB after therapy than those who did not. Binary logistic regression analysis applied separately for each PAB using T2 T as dependent variable and not reaching T2 T at T1 as reference adjusted for sex, smoking, diabetes and T0 proportion of sites with periodontal pockets, showed a significant association between therapeutic outcome and residual bacterial infection; *P. gingivalis* [aOR 1.25 (1.08;1.44), *p* = 0.003], *F. nucleatum* [aOR 1.39(1.11;1.70), *p* = 0.001], *T. forsythia* [aOR 2.39(1.45; 3.94), *p* < 0.001], and *T. denticola* [aOR 1.49 (1.16; 1.92), *p* = 0.002] (Table 5).

**Table 4:** Linear regression model—dependent variable Δpathogen.

Variables	Linear regression		
Independent	β -Coefficient (95% CI) adjusted for T0 pathogen concentration, sex, smoking, diabetes	*p*-value	R Square
*ΔAa*			
Antibiotics (None vs. MET)	−0.08 (−0.41; 0.25)	0.643	0.164
Antibiotics (AMOX + MET vs. MET)	0.41 (−0.12; 0.93)	0.129	
*ΔPg*			
Antibiotics (None vs. MET)	−0.54 (−1.17; 0.08)	0.088	0.363
Antibiotics (AMOX + MET vs. MET)	0.60 (−0.41; 1.61)	0.240	
*ΔFn*			
Antibiotics (None vs. MET)	−0.50 (−1.00; 0.00)	0.082	0.285
Antibiotics (AMOX + MET vs. MET)	−0.24 (−1.06; 0.57)	0.559	
*ΔPi*			
Antibiotics (None vs. MET)	−0.11 (−0.42; 0.20)	0.481	0.322
Antibiotics (AMOX + MET vs. MET)	−0.25 (−0.75; 0.24)	0.314	
*ΔTf*			
Antibiotics (None vs. MET)	−0.25 (−0.47; −0.03)	**0** **.** **026**	0.217
Antibiotics (AMOX + MET vs. MET)	0.01 (−0.35; 0.36)	0.967	
*ΔTd*			
Antibiotics (None vs. MET)	−0.42 (−0.82; −0.03)	**0**.**034**	0.269
Antibiotics (AMOX + MET vs. MET)	0.11 (−0.52; 0.75)	0.727	

AMOX, amoxicillin*; Aa*, *Aggregatibacter actinomycetemcomitans*; *Pg*, *Porphyromonas gingivalis*; *Pi*, *Prevotella intermedia*; *Fn*, *Fusobacterium nucleatum*; MET, metronidazole; T0, prior to steps I and II therapy; *Td*, *Treponema denticola*; *Tf*, *Tannerella forsythia.*

Data are presented as adjusted β-Coefficient with corresponding 95% confidence interval (CI).

Bold indicates statistically significant values (*P* < 0.05).

**Table 5:** Binary logistic regression model—dependent variable T2T at T1.

Variables	Logistic regression—reference not reaching T2T at T1 Adjusted for sex, smoking, diabetes		
Independent	aOR (95% CI)	*p*-value	Nagelkerke *R*^2^
T1			
*Aa*	1.06 (0.81; 1.39)	0.689	0.028
*Pg*	1.25 (1.08; 1.44)	**0** **.** **003**	0.068
*Fn*	1.39 (1.11; 1.70)	**0**.**001**	0.085
*Pi*	1.24 (0.91; 1.71)	0.177	0.047
*Tf*	2.39 (1.45; 3.94)	**<0**.**001**	0.097
*Td*	1.49 (1.16; 1.92)	**0**.**002**	0.080

*Aa*, *Aggregatibacter actinomycetemcomitans*; *Pg*, *Porphyromonas gingivalis*; *Pi*, *Prevotella intermedia*; *Fn*, *Fusobacterium nucleatum*; T0, prior to steps I and II therapy; T1 after steps I and II therapy; T2T, treat-to-target endpoint; *Td*, *Treponema denticola*; *Tf*, *Tannerella forsythia.*

Data are presented as adjusted odds ratio (aOR) with corresponding 95% confidence interval (CI).

Bold indicates statistically significant values (*P* < 0.05).

Considering the overall treatment outcome T2T, apart from the percentage of periodontal pockets at T0, logistic regression analysis using T2T as dependent variable and not reaching T2T at T1 as reference revealed that no antibiotic use in comparison to MET was associated with a 2.4-fold increased risk of not reaching the targeted endpoint [aOR: 2.38 (1.02; 5.53), *p* = 0.044] (Table 6). To verify the results further, a linear regression model was calculated using the proportional reduction of sites with periodontal pockets as dependent variable, which confirmed the outcomes of the binary analysis. In addition to the proportion of sites with periodontal pockets at T0 [β: 0.19 (0.11; 0.67) *p* = 0.006], it was evident that MET as adjuvant compared to no systematic antibiotic resulted in a significant improvement [β: 0.20 (−29.53; −4.45) *p* = 0.008]; however, no distinction was observed between MET and AMOX + MET [β: 0.04 (−13.94; 24.45) *p* = 0.480] (Table 6).

**Table 6:** Regression models for therapy outcome.

Variables	Multivariate binary logistic regression—reference not reaching T2T at T1		Multivariate linear regression—dependent variable proportional reduction of sites with periodontal pockets	
Independent	OR (95% CI)	*p*-value	β -Coefficient (95% CI)	*p*-value
Sex (male vs. female)	1.28 (0.74; 2.22)	0.382	−0.01 (−9.30; 7.96)	0.879
Antibiotics (none vs. MET)	2.38 (1.02; 5.53)	**0** **.** **044**	−0.20 (−29.53; −4.45)	**0**.**008**
Antibiotics (AMOX + MET vs. MET)	0.42 (0.12; 1.46)	0.171	0.04 (−13.94; 24.45)	0.480
Smoking	1.19 (0.62; 2.29)	0.596	−0.12 (−19.44; 0.00)	0.050
Diabetes	0.78 (0.31; 1.96)	0.598	−0.02 (−17.43; 12.12)	0.724
PPD% T0	1.06 (1.04; 1.09)	**<0**.**001**	0.19 (0.11; 0.67)	**0**.**006**
Nagelkerke *R*^2^	0.194		R Square	0.118

AMOX, amoxicillin; *Aa*, *Aggregatibacter actinomycetemcomitans*; *Pg*, *Porphyromonas gingivalis*; *Pi*, *Prevotella intermedia*; *Fn*, *Fusobacterium nucleatum*; MET, metronidazole; PPD%, percentage of sites with probing depth >3 mm; T0, prior to steps I and II therapy; T1 after steps I and II therapy; T2T, treat-to-target endpoint; *Td*, *Treponema denticola*; *Tf*, *Tannerella forsythia.*

Data are presented as odds ratio (OR) or β -Coefficient with corresponding 95% confidence interval (CI).

Bold indicates statistically significant values (*P* < 0.05).

## Discussion

This study aimed to analyse the effect of different antibiotics during non-surgical therapy on the microbial load of selected PAB collected from the subgingival biofilm and the primary therapy outcome. The present study suggests an association between residual levels of selected PAB after steps I and II therapy and the overall treatment outcome. The use of systemic antibiotics was beneficial in PAB reduction but there were no differences found between AMOX + MET and MET alone. Based on these observations, and pending further research, the need for a combined use of AMOX and MET may be questioned.

Determination of the microbial load was done combining DNA amplification with a hybridisation-technique, thus allowing a semi-quantitative analysis (46, 47). Quantitative statements about bacteria are of particular importance when considering the development of disease in the context of critical biomass and help to better understand the disease and the outcome of therapy (33). Nevertheless, there are several other well established methods available i.e., culture-based methods (48), sequencing of 16S ribosomal RNA or shotgun metagenomic sequencing (33, 49, 50). Given the substantial variations in microbial composition within periodontal pockets among individuals (51), the analyses were done pooled per patient to allow conclusions at the patient level.

For the evaluation of the primary treatment outcome, the definition of T2T according to Feres et al. was used (43). This allows a more straightforward comparison of the treatment response at patient level, facilitating future comparisons of results. In studies investigating the beneficial effect of antibiotics, this approach is widely accepted and closely aligns with the clinical results following steps I and II of therapy (5, 42, 43). In our cohort, only 38.2% of patients were successful in achieving the T2 T endpoint. Previous data suggested common success rates of roughly 50% (43). Using a stricter definition of periodontal stability [≤4 mm (no site ≥4 mm with BOP) and BOP <10%], Bertl et al. concluded that fully stable periodontitis following non-surgical therapy may not be achievable for patients with stage III and IV periodontitis. This is consistent with the results of our study, which showed that only 1.1% of patients could meet the criteria of this definition at re-evaluation (52). Moreover, the findings of this study demonstrate that there is no discrepancy in achieving the T2T between patients who have received antibiotics and those who have not. This observation stands in contrast to the results reported by Benz et al., who have noted significant variations in this regard (53). The discrepancy can be attributed to the healthier state of the control group in our study, relative to those in the aforementioned study. In order to address this issue, a linear model was employed, using the proportional reduction of sites with periodontal pockets as the dependent variable. This further proved the findings of the study. However, the study demonstrated only minor effects in terms of the reduction of PAB, particularly the additional effect of antibiosis, which was relatively small and limited to a few species. According to studies by Rams et al. and Jepsen et al., this is not likely to be attributable to the susceptibility of individual species (54, 55). The only notable difference is that *A. actinomycetemcomitans* exhibits only good efficacy on AMOX and not to MET. However, all other species examined here respond in general well to MET (55). Nevertheless, the suboptimal oral hygiene level of the subjects at T1 could provide a possible explanation, in addition to the time interval between T0 and T1. The studies by Lu et al. and Bizzarro et al. demonstrated that the beneficial microbiological effects of antibiotics may level out after 6 months following treatment. As our re-evaluation was after approximately 6 months, this must be considered when interpreting the data (56, 57).

The current results support the concept of a critical threshold of biomass sufficient to induce and maintain an immune response in the periodontal pocket; this individual threshold is highly dependent on numerous genetic, epigenetic and other host-specific factors. This immune response alters the local environmental conditions, driving ecological shifts within the biofilm. These shifts lead to an overgrowth of PAB, ultimately causing irreversible tissue destruction (58–60). As shown herein, steps I and II therapy have the potential to reduce PAB below such a critical threshold leading to short or long-term resolution of periodontitis-associated inflammation, ultimately resulting in treatment success (59, 61). Although the composition of the subgingival microbiome is subjected to dynamic changes, largely determined by external factors such as smoking and oral hygiene practices, this study revealed a clear link between residual microbial load of PAB and treatment outcomes after controlling for confounding factors.

The current findings challenge the therapeutic effects of the combined use of AMOX and MET given the negligible additional clinical improvements over MET alone. This was evident not only when considering the clinical outcomes but also for the microbial level. These results are partially in line with the consensus report from 2020 showing also a significant effect on probing depth reduction by MET alone (16). However, the consensus report emphasised the strong unwanted side effects associated with AMOX + MET. These side effects could potentially be avoided by using MET as a standalone regimen.

Historically, AMOX was included in the antibiotic regimens due to van Winkelhoff's hypothesis that a specific antibiotic was needed to target *A. actinomycetemcomitans*, which is not susceptible to MET (17). Nevertheless, many of the remaining PABs are anaerobic and can therefore be specifically reduced by MET. The early results from Loesche et al. were able to show that MET alone, even in the low dosage of 250 mg, has good and long-term effects on the therapeutic success of non-surgical periodontitis therapy (18–20). This, combined with our results, suggest that the need for AMOX in combination therapy may not be as important as previously proposed.

Moreover, AMOX is one of the antibiotics for which resistance severely increased among PAB in the recent years (27). The current prevalence of AMOX-resistant PAB was recently reported to be 0.4%–1.4% in a cohort of German periodontitis patients (54), which is classified as low by both the European Centre for Disease Prevention and Control and the World Health Organization (62). However, resistance is predicted to rise nearly 28-fold over the next 20 years, potentially contributing to a silent epidemic (21, 27). For this reason, future studies should try to delineate whether the combination of AMOX and MET is truly superior to MET alone. Another interesting new candidate as an antibiotic that could be used as an adjunct for periodontitis treatment is amixicil, which inhibits pyruvate ferredoxin oxidoreductase. This is a mechanism that specifically targets anaerobic bacteria (similarly to MET, which inhibits nucleic acid synthesis by forming nitroso radicals), but leaving a large number of health-associated bacteria untouched (29). Amixicil, which was primarily developed for the extended treatment of *Clostridium difficile* infections, has already been validated for use in periodontitis treatment in animal studies and could serve as potential alternative to MET thereby supporting the question of the necessity of an additional prescription of AMOX (28, 63, 64).

The present study has several limitations that might specifically impair its generalisability, applicability and transferability. As a monocentric observational study, the generalisability of the findings is limited. Also, the rather small AMOX + MET group provides only limited power, which might thus potentially fail to delineate significant effects or differences. Furthermore, this study does not consider the prevalence or potential role of beneficial commensal species. Instead, it follows a targeted approach for a focused analysis of selected PABs most strongly associated with disease progression and response to therapy. Hemoglobin A1c (HbA1c) values were not systematically recorded in the study and therefore not reported. Although this must be considered a limitation, given that only 8.9% of our patients had diabetes, the lack of documentation of HbA1c levels should not affect the general conclusions of this study. Further data on prior systemic antibiotic or oral antiseptics were not collected, leading to potential interference. In the present cohort patients were not specifically selected for this study; rather, they were treated as part of routine clinical training, which was conducted in an undergraduate programme under the supervision of experienced periodontists, which might significantly affect the comparability of data. However, the overall treatment success can be considered satisfactory (10, 65). Additionally, to ensure high quality diagnostic measurements all PPDs were controlled by one of two previously calibrated examiners. Besides these, no patient-related outcome variables were recorded, such as side effects of the antibiotic treatment. Furthermore, the microbial mass increases with the severity of the periodontitis, as does the proportional risk of treatment failure (10, 46). To mitigate this bias, we adjusted the analysis for both T0 PAB level as well as T0 periodontal status.

The rationale for the use of systemic antibiotics in periodontal therapy is matter of debate since many years. Considering AMR, a targeted use of antibiotics in periodontal therapy should be recommended. The results of the present study indicate that the residual microbial load is directly associated with the overall therapy outcome. Furthermore, the findings suggest that antibiotics were more efficient in the reduction of PAB concentrations. However, based on this study, the hypothesis could arise that MET alone could potentially be sufficiently effective as adjunctive to periodontal treatment. To confirm this, further prospective studies with bigger sample size are needed.

## Data Availability

The datasets presented in this article are not readily available because the dataset contains sensitive patient information and is subject to data protection regulations. All personal identifiers have been anonymized, and access is restricted to authorized researchers. Requests to access the datasets should be directed to nils.werner@med.uni-muenchen.de.

## Key finding

• Metronidazole from a clinical point of view might potentially be enough as adjunctive to periodontal treatment.
